# Supplementary material for: Altered microRNA Transcriptome in Cultured Human Liver Cells upon Infection with Ebola Virus
Source: Int J Mol Sci. 2021 Apr 6;22(7):3792. doi: 10.3390/ijms22073792 (PMC8038836; doi:10.3390/ijms22073792)
Supplement: Supplementary file 1 [file ijms-22-03792-s001.zip › Supplementary_File/D_ KEGG_Analysis_Results/16-30nt_pathway_Makona-96h-Huh7_vs_Control-96h-Huh7_down.mature_mirna_targets/hsa_pathwayResult.html]

| PathwayID | Definition | OriginalWebSite | Fisher-Pvalue | SelectionCounts | SelectionSize | Count | Size | FDR | Enrichment\_Score | GeneRatio | Genes |
| --- | --- | --- | --- | --- | --- | --- | --- | --- | --- | --- | --- |
| hsa05030 | Cocaine addiction - Homo sapiens (human) | http://www.genome.jp/kegg-bin/show\_pathway?hsa05030+84699+9586+1742+2904+2912+4790+6531 | 5.410456e-04 | 7 | 234 | 49 | 7906 | 1.645133e-01 | 3.266766 | 0.029915 | CREB3L3//CREB5//DLG4//GRIN2B//GRM2//NFKB1//SLC6A3 |
| hsa05219 | Bladder cancer - Homo sapiens (human) | http://www.genome.jp/kegg-bin/show\_pathway?hsa05219+1029+1613+2261+5894+11186+7057 | 1.189538e-03 | 6 | 234 | 41 | 7906 | 1.645133e-01 | 2.924622 | 0.025641 | CDKN2A//DAPK3//FGFR3//RAF1//RASSF1//THBS1 |
| hsa04921 | Oxytocin signaling pathway - Homo sapiens (human) | http://www.genome.jp/kegg-bin/show\_pathway?hsa04921+9254+786+10645+2002+2775+3768+4776+8605+5532+5562+5564+5894 | 1.886840e-03 | 12 | 234 | 153 | 7906 | 1.645133e-01 | 2.724265 | 0.051282 | CACNA2D2//CACNG1//CAMKK2//ELK1//GNAO1//KCNJ12//NFATC4//PLA2G4C//PPP3CB//PRKAA1//PRKAB1//RAF1 |
| hsa04710 | Circadian rhythm - Homo sapiens (human) | http://www.genome.jp/kegg-bin/show\_pathway?hsa04710+8553+26224+5562+5564+6500 | 1.970219e-03 | 5 | 234 | 31 | 7906 | 1.645133e-01 | 2.705485 | 0.021368 | BHLHE40//FBXL3//PRKAA1//PRKAB1//SKP1 |
| hsa04014 | Ras signaling pathway - Homo sapiens (human) | http://www.genome.jp/kegg-bin/show\_pathway?hsa04014+1944+2002+2263+2261+2904+3815+4790+5062+8605+5894+10928+5900+11186+5966+7074 | 3.636083e-03 | 15 | 234 | 232 | 7906 | 2.428904e-01 | 2.439366 | 0.064103 | EFNA3//ELK1//FGFR2//FGFR3//GRIN2B//KIT//NFKB1//PAK2//PLA2G4C//RAF1//RALBP1//RALGDS//RASSF1//REL//TIAM1 |
| hsa05200 | Pathways in cancer - Homo sapiens (human) | http://www.genome.jp/kegg-bin/show\_pathway?hsa05200+613+840+8030+1027+1029+1613+1906+2002+2263+2261+2535+7976+26508+3162+3566+3815+4790+7849+5292+5371+5894+10928+5900+11186+6500+7481 | 7.145678e-03 | 26 | 234 | 529 | 7906 | 3.514001e-01 | 2.145957 | 0.111111 | BCR//CASP7//CCDC6//CDKN1B//CDKN2A//DAPK3//EDN1//ELK1//FGFR2//FGFR3//FZD2//FZD3//HEYL//HMOX1//IL4R//KIT//NFKB1//PAX8//PIM1//PML//RAF1//RALBP1//RALGDS//RASSF1//SKP1//WNT11 |
| hsa04360 | Axon guidance - Homo sapiens (human) | http://www.genome.jp/kegg-bin/show\_pathway?hsa04360+1944+7976+4776+5062+84612+5532+5894+56963+57715+54437+8482+90249 | 7.364674e-03 | 12 | 234 | 181 | 7906 | 3.514001e-01 | 2.132846 | 0.051282 | EFNA3//FZD3//NFATC4//PAK2//PARD6B//PPP3CB//RAF1//RGMA//SEMA4G//SEMA5B//SEMA7A//UNC5A |
| hsa04916 | Melanogenesis - Homo sapiens (human) | http://www.genome.jp/kegg-bin/show\_pathway?hsa04916+84699+1906+2535+7976+2775+3815+5894+7481 | 9.928016e-03 | 8 | 234 | 101 | 7906 | 4.144947e-01 | 2.003138 | 0.034188 | CREB3L3//EDN1//FZD2//FZD3//GNAO1//KIT//RAF1//WNT11 |
| hsa04390 | Hippo signaling pathway - Homo sapiens (human) | http://www.genome.jp/kegg-bin/show\_pathway?hsa04390+1742+2535+7976+4771+84612+11186+4086+7003+7481+7529 | 1.587928e-02 | 10 | 234 | 154 | 7906 | 4.838707e-01 | 1.799169 | 0.042735 | DLG4//FZD2//FZD3//NF2//PARD6B//RASSF1//SMAD1//TEAD1//WNT11//YWHAB |
| hsa04725 | Cholinergic synapse - Homo sapiens (human) | http://www.genome.jp/kegg-bin/show\_pathway?hsa04725+1128+1136+84699+9586+2775+3768+9132+56479 | 1.774327e-02 | 8 | 234 | 112 | 7906 | 4.838707e-01 | 1.750966 | 0.034188 | CHRM1//CHRNA3//CREB3L3//CREB5//GNAO1//KCNJ12//KCNQ4//KCNQ5 |
| hsa05020 | Prion diseases - Homo sapiens (human) | http://www.genome.jp/kegg-bin/show\_pathway?hsa05020+712+2002+3553+4684 | 1.907722e-02 | 4 | 234 | 35 | 7906 | 4.838707e-01 | 1.719485 | 0.017094 | C1QA//ELK1//IL1B//NCAM1 |
| hsa04122 | Sulfur relay system - Homo sapiens (human) | http://www.genome.jp/kegg-bin/show\_pathway?hsa04122+9054+81605 | 2.171149e-02 | 2 | 234 | 8 | 7906 | 4.838707e-01 | 1.663310 | 0.008547 | NFS1//URM1 |
| hsa04015 | Rap1 signaling pathway - Homo sapiens (human) | http://www.genome.jp/kegg-bin/show\_pathway?hsa04015+1944+2263+2261+2775+2904+3815+84612+5894+5900+7057+7074+7094 | 2.188748e-02 | 12 | 234 | 210 | 7906 | 4.838707e-01 | 1.659804 | 0.051282 | EFNA3//FGFR2//FGFR3//GNAO1//GRIN2B//KIT//PARD6B//RAF1//RALGDS//THBS1//TIAM1//TLN1 |
| hsa04550 | Signaling pathways regulating pluripotency of stem cells - Homo sapiens (human) | http://www.genome.jp/kegg-bin/show\_pathway?hsa04550+1749+2263+2261+2535+7976+5894+6498+4086+7481 | 2.289090e-02 | 9 | 234 | 140 | 7906 | 4.838707e-01 | 1.640337 | 0.038462 | DLX5//FGFR2//FGFR3//FZD2//FZD3//RAF1//SKIL//SMAD1//WNT11 |
| hsa05212 | Pancreatic cancer - Homo sapiens (human) | http://www.genome.jp/kegg-bin/show\_pathway?hsa05212+9459+1029+4790+5894+10928+5900 | 2.323794e-02 | 6 | 234 | 75 | 7906 | 4.838707e-01 | 1.633802 | 0.025641 | ARHGEF6//CDKN2A//NFKB1//RAF1//RALBP1//RALGDS |
| hsa05215 | Prostate cancer - Homo sapiens (human) | http://www.genome.jp/kegg-bin/show\_pathway?hsa05215+1027+84699+9586+2263+4790+5894+7113 | 2.456412e-02 | 7 | 234 | 97 | 7906 | 4.838707e-01 | 1.609699 | 0.029915 | CDKN1B//CREB3L3//CREB5//FGFR2//NFKB1//RAF1//TMPRSS2 |
| hsa05133 | Pertussis - Homo sapiens (human) | http://www.genome.jp/kegg-bin/show\_pathway?hsa05133+712+840+3553+3678+4790+114609 | 2.462815e-02 | 6 | 234 | 76 | 7906 | 4.838707e-01 | 1.608568 | 0.025641 | C1QA//CASP7//IL1B//ITGA5//NFKB1//TIRAP |
| hsa04530 | Tight junction - Homo sapiens (human) | http://www.genome.jp/kegg-bin/show\_pathway?hsa04530+10097+10096+84952+83700+4771+84612+5562+5564+7074+7454 | 2.825327e-02 | 10 | 234 | 169 | 7906 | 5.242550e-01 | 1.548931 | 0.042735 | ACTR2//ACTR3//CGNL1//JAM3//NF2//PARD6B//PRKAA1//PRKAB1//TIAM1//WAS |
| hsa00561 | Glycerolipid metabolism - Homo sapiens (human) | http://www.genome.jp/kegg-bin/show\_pathway?hsa00561+10554+1608+132158+253558+80168 | 3.381662e-02 | 5 | 234 | 61 | 7906 | 5.378716e-01 | 1.470870 | 0.021368 | AGPAT1//DGKG//GLYCTK//LCLAT1//MOGAT2 |
| hsa04662 | B cell receptor signaling pathway - Homo sapiens (human) | http://www.genome.jp/kegg-bin/show\_pathway?hsa04662+933+973+975+4790+5532+5894 | 3.414083e-02 | 6 | 234 | 82 | 7906 | 5.378716e-01 | 1.466726 | 0.025641 | CD22//CD79A//CD81//NFKB1//PPP3CB//RAF1 |
| hsa04962 | Vasopressin-regulated water reabsorption - Homo sapiens (human) | http://www.genome.jp/kegg-bin/show\_pathway?hsa04962+397+84699+9586+1780 | 4.030747e-02 | 4 | 234 | 44 | 7906 | 5.378716e-01 | 1.394614 | 0.017094 | ARHGDIB//CREB3L3//CREB5//DYNC1I1 |
| hsa04931 | Insulin resistance - Homo sapiens (human) | http://www.genome.jp/kegg-bin/show\_pathway?hsa04931+84699+9586+4790+8473+5562+5564+10999 | 4.073733e-02 | 7 | 234 | 108 | 7906 | 5.378716e-01 | 1.390007 | 0.029915 | CREB3L3//CREB5//NFKB1//OGT//PRKAA1//PRKAB1//SLC27A4 |
| hsa05221 | Acute myeloid leukemia - Homo sapiens (human) | http://www.genome.jp/kegg-bin/show\_pathway?hsa05221+3815+4790+5292+5371+5894 | 4.518673e-02 | 5 | 234 | 66 | 7906 | 5.378716e-01 | 1.344989 | 0.021368 | KIT//NFKB1//PIM1//PML//RAF1 |
| hsa04310 | Wnt signaling pathway - Homo sapiens (human) | http://www.genome.jp/kegg-bin/show\_pathway?hsa04310+1501+8061+2535+7976+4776+5532+6422+6500+7481 | 4.771744e-02 | 9 | 234 | 160 | 7906 | 5.378716e-01 | 1.321323 | 0.038462 | CTNND2//FOSL1//FZD2//FZD3//NFATC4//PPP3CB//SFRP1//SKP1//WNT11 |
| hsa04211 | Longevity regulating pathway - Homo sapiens (human) | http://www.genome.jp/kegg-bin/show\_pathway?hsa04211+10645+84699+9586+4790+5562+5564 | 4.790707e-02 | 6 | 234 | 89 | 7906 | 5.378716e-01 | 1.319600 | 0.025641 | CAMKK2//CREB3L3//CREB5//NFKB1//PRKAA1//PRKAB1 |
| hsa04668 | TNF signaling pathway - Homo sapiens (human) | http://www.genome.jp/kegg-bin/show\_pathway?hsa04668+840+84699+9586+1906+3553+4790+10454 | 4.801499e-02 | 7 | 234 | 112 | 7906 | 5.378716e-01 | 1.318623 | 0.029915 | CASP7//CREB3L3//CREB5//EDN1//IL1B//NFKB1//TAB1 |
